# Special Issue “New Molecular Insights into Ischemia/Reperfusion”

**DOI:** 10.3390/ijms26010212

**Published:** 2024-12-30

**Authors:** Ji Hyeon Ahn, Moo-Ho Won

**Affiliations:** 1 Department of Physical Therapy, College of Health Science, Youngsan University, Yangsan 50510, Republic of Korea; jh-ahn@ysu.ac.kr; 2 Department of Emergency Medicine, Kangwon National University Hospital, Chuncheon 24289, Republic of Korea

Ischemia/reperfusion (IR) injury is a complex pathophysiological process in which the restoration of blood flow to ischemic tissue paradoxically exacerbates tissue damage and death [1]. IR injury can occur in various organs, including the heart, brain, spinal cord, liver, and kidneys, during cardiac arrest, ischemic stroke, and organ transplantation. Studies have shown that oxidative stress, inflammatory responses, and excitotoxicity are the main mechanisms of IR injury [2,3,4]; however, the detailed molecular mechanisms for treating IR injury in various organs are still limited, and there are no definitive treatments. This Special Issue “New Molecular Insights into Ischemia/Reperfusion” presents the latest molecular mechanisms of IR damage in specific organs (brain, heart, and kidney) and proposes a broad range of strategies to mitigate and treat IR damage. A molecular understanding of IR injury in each organ is an important foundation for developing potential therapeutics and clinical applications.

Multifactorial cerebral IR pathogenesis involves several mechanisms, which include key molecules like various inflammatory mediators that induce signaling via pattern recognition receptors such as Toll-like receptors (TLRs) and immune cell activation, and reactive oxygen species (ROS) that directly damage cell and organelle membranes through lipid peroxidation [5]. Danger-associated molecular patterns (DAMPs) such as peroxiredoxins released after reperfusion are known to bind to TLRs on microglia and leukocytes, activate inflammatory transcription factors such as nuclear factor-κB (NF-κB) and mitogen-activated protein kinase (MAPK) [6,7], and produce cytokines and chemokines, thereby aggravating ischemic damage [8]. In this Special Issue, one team proposes a preconditioning approach with a compound (aucubin, an iridoid glycoside, a type of bioactive compound found in traditional herbal medicine) targeting TRL4/NF-κB to alleviate ischemic brain damage [9], while another team proposes an early postconditioning approach with therapeutic electrical stimulation (high-definition-transcranial alternating current stimulation (HD-tACS), high-definition-transcranial direct current stimulation (HD-tDCS), or electroacupuncture (EA)) that regulates the signal transducer and activator of transcription 1 (STAT1) and NF-kB activation to enhance neuronal survival and alleviate motor dysfunction [10]. In a review paper [11], the development of hypoxia-inducible factors (HIF)-1α inhibitors is proposed, which has not been clinically tested in stroke. Under severe hypoxic conditions, the imbalanced accumulation of HIF-1α induces various gene expression changes and post-translational modifications related to cellular lipid peroxidation [12], and HIF-1α-mediated pathways include neurovascular inflammation, glial cell activation, and apoptosis [13,14]. Therefore, appropriate inhibition of HIF-1α may alleviate cell damage, so the development of appropriate HIF-1α inhibition techniques during severe hypoxia in stroke may have sufficient clinical value.

Regarding cardiac IR injury, ferroptosis has emerged as a target for the development of novel drugs to prevent myocardial infarction due to cardiac IR injury [15]. Ferroptosis is a type of cell death induced by oxidative stress, where an iron-dependent nonapoptotic cell death process induces cell membrane rupture and cell death through lipid peroxidation [16]. Reperfusion after coronary artery occlusion (CAO) induces cardiac damage and myocardial infarction [17,18] through myocardial ferroptosis [19,20,21,22]. After IR cardiac injury, malondialdehyde (MDA), Fe^2+^, and ferritin heavy chain-1 (FTH1) levels are increased [23], while glutathione peroxidase-4 (GPX4) expression is decreased [23,24]. Previous studies show that miR-15a-5p induces IR cardiac injury by activating ferroptosis, and ferrostatin-1, lipostatin-1, and antioxidant histochrome inhibit ferroptosis and reduce infarct size [17,24,25,26]. Taken together, ferroptosis inhibitors such as ferroptosis-1 and lipoxanthin-1 have cardioprotective effects, but their low water solubility makes intravenous administration impossible, so the development of ferroptosis inhibitors with high solubility is necessary. Drug development to control ferroptosis could be utilized as effective treatments for acute myocardial infarction.

In terms of renal IR injury, renal IR occurs inevitably during renal transplantation and surgery, which can cause acute tubular necrosis, which can lead to acute kidney injury (AKI), a serious clinical syndrome that shows structural and functional damage to the kidney within a short period of time [27]. Renal IR-induced disruption of oxygen metabolism can generate excessive ROS, inducing nonspecific oxidation of cardiolipin, a mitochondrial phospholipid that plays an important role in mitochondrial metabolism, and increasing cardiolipin content through pathological remodeling and resynthesis mechanisms, which can further aggravate damage to mitochondria and cells [28]. In human renal proximal tubule epithelial cells (RPTECs), hypoxia/reoxygenation increases mRNA and protein expression of cardiolipin synthase and lysocardiolipin acyltransferase 1 (LCLAT1), a remodeling enzyme [29], followed by increased cardiolipin levels during hypoxia [30]. LCLAT1 overexpression is involved in pathological remodeling of cardiolipin, which impairs mitochondrial functions and leads to further cellular damage. Although IR-induced changes in cardiolipin content and oxidation and/or hydrolysis of cardiolipins are reported in brain and heart tissue [28,31], few studies are reported in the kidney, so results of cardiolipin studies in the kidney are noteworthy.

IR injury can be managed by preconditioning before or during blood flow interruption, or by postconditioning after reperfusion. Preconditioning strategies are useful in clinically predictable ischemic events such as organ transplantation, while postconditioning has greater clinical applicability in unpredictable events such as cardiac arrest or stroke. Although IR injury in each organ has its own unique characteristics in different complex situations, the production of stress mediators occurring as a result of energy depletion, oxidative stress, and inflammatory responses share diverse cellular mechanisms and pathways between organs. To overcome organ-specific IR injury, advanced molecular mechanisms associated with IR injury need to be continuously studied. In order to improve the basic knowledge of organ-specific IR injury management and enable clinical application, in-depth studies of complex molecular mechanisms that reflect the specificity of each organ are needed, and the results should be standardized in a clinically applicable manner.

## References

[1] Zhang M., Liu Q., Meng H., Duan H., Liu X., Wu J., Gao F., Wang S., Tan R., Yuan J. (2024). Ischemia-reperfusion injury: Molecular mechanisms and therapeutic targets. Signal Transduct. Target. Ther..

[2] He J., Liu D., Zhao L., Zhou D., Rong J., Zhang L., Xia Z. (2022). Myocardial ischemia/reperfusion injury: Mechanisms of injury and implications for management. Exp. Ther. Med..

[3] Zhang Q., Jia M., Wang Y., Wang Q., Wu J. (2022). Cell death mechanisms in cerebral ischemia–reperfusion injury. Neurochem. Res..

[4] Han S.J., Lee H.T. (2019). Mechanisms and therapeutic targets of ischemic acute kidney injury. Kidney Res. Clin. Pract..

[5] Naito H., Nojima T., Fujisaki N., Tsukahara K., Yamamoto H., Yamada T., Aokage T., Yumoto T., Osako T., Nakao A. (2020). Therapeutic strategies for ischemia reperfusion injury in emergency medicine. Acute Med. Surg..

[6] Abe J.-i., Baines C.P., Berk B.C. (2000). Role of mitogen-activated protein kinases in ischemia and reperfusion injury: The good and the bad. Circ. Res..

[7] Harari O.A., Liao J.K. (2010). NF-κB and innate immunity in ischemic stroke. Ann. N. Y. Acad. Sci..

[8] Danton G.H., Dietrich W.D. (2003). Inflammatory mechanisms after ischemia and stroke. J. Neuropathol. Exp. Neurol..

[9] Park J.H., Lee T.-K., Kim D.W., Ahn J.H., Shin M.C., Cho J.H., Won M.-H., Kang I.J. (2024). Neuroprotective Effects of Aucubin against Cerebral Ischemia and Ischemia Injury through the Inhibition of the TLR4/NF-κB Inflammatory Signaling Pathway in Gerbils. Int. J. Mol. Sci..

[10] Lee H., Lee J., Jung D., Oh H., Shin H., Choi B. (2024). Neuroprotection of Transcranial Cortical and Peripheral Somatosensory Electrical Stimulation by Modulating a Common Neuronal Death Pathway in Mice with Ischemic Stroke. Int. J. Mol. Sci..

[11] Choi Y.K. (2024). Detrimental Roles of Hypoxia-Inducible Factor-1α in Severe Hypoxic Brain Diseases. Int. J. Mol. Sci..

[12] Song W., Chen Y., Qin L., Xu X., Sun Y., Zhong M., Lu Y., Hu K., Wei L., Chen J. (2023). Oxidative stress drives vascular smooth muscle cell damage in acute Stanford type A aortic dissection through HIF-1α/HO-1 mediated ferroptosis. Heliyon.

[13] Jung E., Kim Y.E., Jeon H.S., Yoo M., Kim M., Kim Y.-M., Koh S.-H., Choi Y.K. (2023). Chronic hypoxia of endothelial cells boosts HIF-1α-NLRP1 circuit in Alzheimer’s disease. Free Radic. Biol. Med..

[14] Yuan D., Guan S., Wang Z., Ni H., Ding D., Xu W., Li G. (2021). HIF-1α aggravated traumatic brain injury by NLRP3 inflammasome-mediated pyroptosis and activation of microglia. J. Chem. Neuroanat..

[15] Ryabov V.V., Maslov L.N., Vyshlov E.V., Mukhomedzyanov A.V., Kilin M., Gusakova S.V., Gombozhapova A.E., Panteleev O.O. (2024). Ferroptosis, a Regulated Form of Cell Death, as a Target for the Development of Novel Drugs Preventing Ischemia/Reperfusion of Cardiac Injury, Cardiomyopathy and Stress-Induced Cardiac Injury. Int. J. Mol. Sci..

[16] Dixon S.J., Lemberg K.M., Lamprecht M.R., Skouta R., Zaitsev E.M., Gleason C.E., Patel D.N., Bauer A.J., Cantley A.M., Yang W.S. (2012). Ferroptosis: An iron-dependent form of nonapoptotic cell death. Cell.

[17] Yu P., Zhang J., Ding Y., Chen D., Sun H., Yuan F., Li S., Li X., Yang P., Fu L. (2022). Dexmedetomidine post-conditioning alleviates myocardial ischemia–reperfusion injury in rats by ferroptosis inhibition via SLC7A11/GPX4 axis activation. Hum. Cell.

[18] Li S., Lei Z., Yang X., Zhao M., Hou Y., Wang D., Tang S., Li J., Yu J. (2022). Propofol protects myocardium from ischemia/reperfusion injury by inhibiting ferroptosis through the AKT/p53 signaling pathway. Front. Pharmacol..

[19] Liu X., Qi K., Gong Y., Long X., Zhu S., Lu F., Lin K., Xu J. (2022). Ferulic acid alleviates myocardial ischemia reperfusion injury via upregulating AMPKα2 expression-mediated ferroptosis depression. J. Cardiovasc. Pharmacol..

[20] Miyamoto H.D., Ikeda M., Ide T., Tadokoro T., Furusawa S., Abe K., Ishimaru K., Enzan N., Sada M., Yamamoto T. (2022). Iron overload via heme degradation in the endoplasmic reticulum triggers ferroptosis in myocardial ischemia-reperfusion injury. Basic Transl. Sci..

[21] Jiang Y.-Q., Yang X.-Y., Duan D.-Q., Zhang Y.-Y., Li N.-S., Tang L.-J., Peng J., Luo X.-J. (2023). Inhibition of MALT1 reduces ferroptosis in rat hearts following ischemia/reperfusion via enhancing the Nrf2/SLC7A11 pathway. Eur. J. Pharmacol..

[22] Zhang Z., Jiang W., Zhang C., Yin Y., Mu N., Wang Y., Yu L., Ma H. (2023). Frataxin inhibits the sensitivity of the myocardium to ferroptosis by regulating iron homeostasis. Free Radic. Biol. Med..

[23] Yang F., Wang W., Zhang Y., Nong J., Zhang L. (2023). Effects of ferroptosis in myocardial ischemia/reperfusion model of rat and its association with Sestrin 1. Adv. Clin. Exp. Med..

[24] Feng Y., Madungwe N.B., Aliagan A.D.I., Tombo N., Bopassa J.C. (2019). Ferroptosis inhibitor, liproxstatin-1, protects the myocardium against ischemia/reperfusion injury by decreasing VDAC1 levels and rescuing GPX4 levels. Biochem. Biophys. Res. Commun..

[25] Fang X., Wang H., Han D., Xie E., Yang X., Wei J., Gu S., Gao F., Zhu N., Yin X. (2019). Ferroptosis as a target for protection against cardiomyopathy. Proc. Natl. Acad. Sci. USA.

[26] Hwang J.-W., Park J.-H., Park B.-W., Kim H., Kim J.-J., Sim W.-S., Mishchenko N.P., Fedoreyev S.A., Vasileva E.A., Ban K. (2021). Histochrome attenuates myocardial ischemia-reperfusion injury by inhibiting ferroptosis-induced cardiomyocyte death. Antioxidants.

[27] Turgut F., Awad A.S., Abdel-Rahman E.M. (2023). Acute kidney injury: Medical causes and pathogenesis. J. Clin. Med..

[28] Paradies G., Paradies V., Ruggiero F.M., Petrosillo G. (2018). Mitochondrial bioenergetics and cardiolipin alterations in myocardial ischemia-reperfusion injury: Implications for pharmacological cardioprotection. Am. J. Physiol.-Heart Circ. Physiol..

[29] Jang S., Javadov S. (2023). Unraveling the mechanisms of cardiolipin function: The role of oxidative polymerization of unsaturated acyl chains. Redox Biol..

[30] Strazdauskas A., Trumbeckaite S., Jakstas V., Dambrauskiene J., Mieldazyte A., Klimkaitis K., Baniene R. (2024). In Vitro Hypoxia/Reoxygenation Induces Mitochondrial Cardiolipin Remodeling in Human Kidney Cells. Int. J. Mol. Sci..

[31] Ji J., Baart S., Vikulina A.S., Clark R.S., Anthonymuthu T.S., Tyurin V.A., Du L., St Croix C.M., Tyurina Y.Y., Lewis J. (2015). Deciphering of mitochondrial cardiolipin oxidative signaling in cerebral ischemia-reperfusion. J. Cereb. Blood Flow Metab..

